# Head Transcriptomes of Two Closely Related Species of Fruit Flies of the *Anastrepha fraterculus* Group Reveals Divergent Genes in Species with Extensive Gene Flow

**DOI:** 10.1534/g3.116.030486

**Published:** 2016-08-23

**Authors:** Victor Borges Rezende, Carlos Congrains, André Luís A. Lima, Emeline Boni Campanini, Aline Minali Nakamura, Janaína Lima de Oliveira, Samira Chahad-Ehlers, Iderval Sobrinho Junior, Reinaldo Alves de Brito

**Affiliations:** Departamento de Genética e Evolução, Universidade Federal de São Carlos, São Paulo 13565-905, Brazil

**Keywords:** RNA-Seq, *de novo* assembly, next generation sequencing, fixed SNPs, *fraterculus* group, positive selection

## Abstract

Several fruit flies species of the *Anastrepha fraterculus* group are of great economic importance for the damage they cause to a variety of fleshy fruits. Some species in this group have diverged recently, with evidence of introgression, showing similar morphological attributes that render their identification difficult, reinforcing the relevance of identifying new molecular markers that may differentiate species. We investigated genes expressed in head tissues from two closely related species: *A. obliqua* and *A. fraterculus*, aiming to identify fixed single nucleotide polymorphisms (SNPs) and highly differentiated transcripts, which, considering that these species still experience some level of gene flow, could indicate potential candidate genes involved in their differentiation process. We generated multiple libraries from head tissues of these two species, at different reproductive stages, for both sexes. Our analyses indicate that the *de novo* transcriptome assemblies are fairly complete. We also produced a hybrid assembly to map each species’ reads, and identified 67,470 SNPs in *A. fraterculus*, 39,252 in *A. obliqua*, and 6386 that were common to both species. We identified 164 highly differentiated unigenes that had a mean interspecific index ($\bar{D}$) of at least 0.94. We selected unigenes that had Ka/Ks higher than 0.5, or had at least three or more highly differentiated SNPs as potential candidate genes for species differentiation. Among these candidates, we identified proteases, regulators of redox homeostasis, and an odorant-binding protein (*Obp99c*), among other genes. The head transcriptomes described here enabled the identification of thousands of genes hitherto unavailable for these species, and generated a set of candidate genes that are potentially important to genetically identify species and understand the speciation process in the presence of gene flow of *A. obliqua* and *A. fraterculus*.

*Anastrepha* is a Neotropical genus distributed from southern regions of the United States to South America, except in Chile (Malavasi *et al.* 2000; Fu *et al.* 2014). These flies are economically important pests because they inflict serious damage caused by oviposition and larval growth on a wide variety of fruits. The genus is comprised of 237 species, which are morphologically divided into 18 species groups (Norrbom *et al.* 1998, 1999; Norrbom and Korytkowski 2009, 2011), though the most common and relevant is the *fraterculus* group, composed of 29 species, some of them cryptic owing to their morphological and genetic similarities (Norrbom *et al.* 1999; Araujo 1997; Zucchi 2000a, 2000b). *A. obliqua* and *A. fraterculus* are closely related species of the *fraterculus* group, and two of the most economically important species in the genus, because of their broad distribution and wide host range (Zucchi 2000a; Solferini and Morgante 1987). In spite of their relevance, there is a lack of evolutionary and genetic studies not only on these species, but on other species of the *fraterculus* group as well. Traditional taxonomy is complicated by an overlap of certain morphological attributes (Zucchi 2000a, 2000b), even though there are some differences in host preferences and reproductive behavior (Aluja 1994; Sivinski *et al.* 1999; Aluja *et al.* 1999), which may indicate early stages of speciation in phytophagous insects (Malavasi and Morgante 1982; Feder *et al.* 1998). Molecular studies performed to date using mtDNA (Smith-Caldas *et al.* 2001; McPheron *et al.* 1999) or nuclear markers (Ruiz *et al.* 2007; Sarno *et al.* 2010; Sobrinho and de Brito 2010, 2012) have also failed to identify species-specific markers.

We seek to understand genes involved with the speciation process among species of *fraterculus* group, aiming at identifying candidate genes and evolutionary forces involved with their differentiation. This is particularly relevant because a recent investigation on 20 different genes has indicated that three species of this group, two of them *A. fraterculus* and *A. obliqua*, have evolved as independent lineages despite evidence of significant introgression, but failed to find fixed specific differences (F. Diaz, A. L. A. Lima, A. M. Nakamura, F. Fernandes, I. Sobrinho, and R. A. de Brito, unpublished results). Using *A. obliqua* and *A. fraterculus* head transcriptome data, we looked for rapidly evolving genes and potential species-specific single nucleotide polymorphisms (SNPs) that would have a better chance of tracking the groups’ differentiation even in the presence of gene flow. These two species are interesting because they show important ecological and behavioral differences. Though somewhat generalists, *A. obliqua* has been more associated with fruits from Anacardiaceae trees, whereas *A. fraterculus* is more commonly found in Myrtaceae (Norrbom *et al.* 1999), which suggests that they could be attracted to different odors from these fruits. These species also show differences in mating time and other reproductive activities (Henning and Matioli 2006; Aluja *et al.* 1999).

There is limited genetic data available for *A. obliqua* and *A. fraterculus*, so we used next generation sequencing technology to generate head transcriptomes of these species. RNA-seq strategies, such as *de novo* transcript determination (Gompert *et al.* 2010; Davey and Blaxter 2010; Robertson *et al.* 2010; Wang *et al.* 2009), and the identification of coding SNPs (Nielsen *et al.* 2011; Schwarz *et al.* 2010; Cánovas *et al.* 2010), have provided a powerful tool with which to identify new sequences that may be useful for genetic as well as evolutionary studies. Studies on transcriptomes from nonmodel organisms are becoming more achievable and computationally tractable, even more so than genome projects (Kumar and Blaxter 2010), although a high computational effort is still required (Schwartz and Waterman 2010). The choice of head tissues owes to the fact that some key genes potentially involved in the determination of interspecific differences, for instance chemoreception and circadian genes, are expressed in the cephalic region because of both ecological and reproductive factors.

This is the first study to investigate head tissue transcriptomes in *Anastrepha*, and to consider samples at different sexes and reproductive stages, which enabled us to identify thousands of SNPs that segregate within and between species, and allowed us to find a set of genes that showed fixed differences between *A. fraterculus* and *A. obliqua*, some of which could be potentially involved in differentiation of these two closely related species.

## Materials and Methods

### Samples

Flies were obtained in the field from guava (Myrtaceae) and jocote (Anacardiaceae) fruits, collected, respectively, in southeastern (22° 01′ 03″ S, 47° 53′ 27″ W) and midwest (16° 41′ 58″ S, 49° 16′ 35″ W) regions of Brazil. Populations of the two species used in this study have been maintained in the Population Genetics and Evolution laboratory at the Federal University of São Carlos, Brazil, for over a year now, in a controlled environment room at 26 ± 5° (60–90% humidity), and natural photoperiod. The newly emerged adults were identified morphologically as *A. obliqua* or *A. fraterculus*, and their descendants were kept in acrylic cages supplied with water and a mixture of hydrolyzed protein, vitamins, and dry sucrose as food. Flies were allowed to mate after they became sexually mature, and their proliferation was performed using mango fruits introduced into the cages for oviposition. The choice of mango as support is due to the fact that both species have commonly been associated with this fruit, and its year round availability. Each fruit was then transferred to a new cage filled with vermiculite into which mature larvae migrated and pupated. Finally, pupae were sieved from the vermiculite and transferred to a new cage, establishing a new generation. In order to reduce inbreeding, these populations were maintained at a size of at least 100 mating adults per generation.

Different transcriptome profiles were generated for different sexes and reproductive stages (virgin and postmating adults of both sexes, and postoviposition females), totaling five different profiles for each species, with replication. Fly heads from different pools of 10 individuals per profile were used for each library, adding up to 100 individuals per species. Virgin flies were killed and had their heads collected 10 d after emergence to ensure they were sexually mature. Pools from virgin flies were produced from flies collected at two different times, at noon and midnight, and combined to enable the identification of genes that might be affected by the circadian clock. Postmating males and females were collected between 15 and 20 hr after a successful copulation, while heads from postoviposition females were collected immediately after a successful oviposition.

### RNA extraction and cDNA library construction

Total RNA was extracted from pools of five heads using the Trizol/chloroform protocol (Chomczynski and Mackey 1995). RNA quality was inspected visually in agarose gels and quantified in both a Qubit fluorometer and a Nanodrop spectrophotometer. For each virgin profile, we combined two pools (one collected at night, and other in the day) per treatment equimolarly with good quality RNA to make a final pool of 10 individuals. RNA-seq libraries were constructed individually for each contrast using the TruSeq RNA Sample Prep kit (Illumina) protocol according to the manufacturer’s instructions. Libraries were sequenced on an Illumina HiSeq2000 at the Laboratory of Functional Genomics Applied to Agriculture and Agri-energy, ESALQ-USP, Brazil, on flow cells with runs of 2 × 100 bp paired-end reads. We used Illumina’s HiSeq Control Software and CASAVA v1.8.2 software (Illumina) for base calling and sample demultiplexing.

### Quality control and de novo assembly

All reads were trimmed for quality and length using the software SeqyClean (Zhbannikov *et al.* 2014). We set “max-avg-error” of 0.01 and “max-error-at-ends” of 0.05 as the trimming parameters, and we kept reads with minimum sequence length of 50 bases. SeqyClean also scans and removes any remaining adapter sequences from the sequences, and analyzes the two complementary sequences (paired-end) together and discards both sequences if one fails to fulfill the filtering requirements established. This feature is important for a more efficient *de novo* assembly, because all reads are paired.

**Figure 1:**
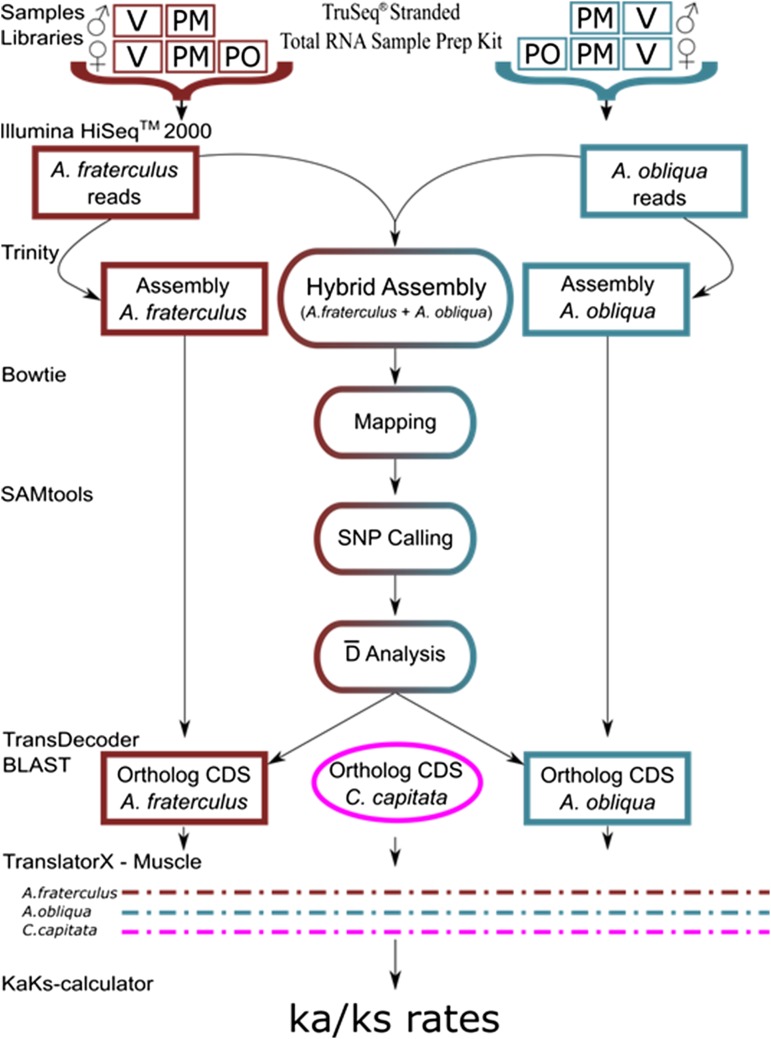
Framework for identifying candidate genes and estimating Ka/Ks in transcriptome libraries of *A. fraterculus* and *A. obliqua*. Separate pool libraries were built from virgin (V) and postmating (PM) males and females, and postovipositing (PO) females, with replicates. The derived reads were combined into single assemblies per species that were investigated for genes potentially involved with species divergence.

Processed reads were assembled using the Trinity short read assembler (release 2013-02-25) (Grabherr *et al.* 2011), using default parameters (kmer length of 25, min_contig_length, min_kmer_cov = 1, max_reads_per_graph = 200,000, max_number_of_paths_per_node = 10, and group_pairs_distance = 500) on a Dell T610 server (24 cores, 128 G of RAM memory) at the Population Genetics and Evolution Laboratory at the Federal University of São Carlos. We performed three assemblies: (i) a hybrid assembly that included all 20 libraries from both species; (ii) an assembly of the 10 libraries from *A. fraterculus*, and (iii) an assembly of the 10 libraries from *A. obliqua*. The hybrid assembly was used as a reference transcriptome for the discovery and comparison of SNPs in both species, to try to reduce the impact of SNP ascertainment bias (McTavish and Hillis 2015). The latter two assemblies were used for orthology determination and screening for potential genes under positive selection (Figure 1).

### Functional annotation

In order to annotate the assembled transcripts, we first evaluated the six open reading frames (ORFs) and predicted the coding sequences (CDSs) using the TransDecoder software included in the Trinity Package, retaining CDSs longer than 100 residues. We used the complete CDSs of each species’ assembly to determine the codon usage tables using the program Cusp (http://emboss.sourceforge.net/apps/cvs/emboss/apps/cusp.html) and to estimate Frank Wright’s Nc statistics using Chips (http://emboss.sourceforge.net/apps/cvs/emboss/apps/chips.html). We compared the predicted CDSs using the BLAST tool in the package “Standalone BLAST Setup for Unix” (Altschul *et al.* 1997) against a set of 248 core eukaryotic genes (CEGs) (Parra *et al.* 2007), *Drosophila melanogaster* CDS database (Dmel R6.02), and CDSs of *Ceratitis capitata* available from the NCBI (20,622 sequences) and cluster of eukaryotic orthologous groups (KOG) databases (Tatusov *et al.* 2003; Koonin *et al.* 2004). Best hits against the KOG database were used to classify the CDSs of the head transcriptomes. Similarity was deemed significant if the *e*-value was ≤ 10^−6^. We used all contigs in each assembly to assess the transcriptome completeness against a set of 2675 almost universal single-copy arthropod orthologs using the software Benchmarking Universal Single-Copy Orthologs (BUSCO) (Simão *et al.* 2015). Best hits against the COG database were used to classify the CDSs of the head transcriptomes. We used the annotation against *D. melanogaster* CDS database to map the gene ontology (GO) terms of the CDSs in categories with the PANTHER classification gene system (GO database release 2015-08-06) (Mi *et al.* 2013; The Gene Ontology Consortium 2013). We performed two overrepresentation tests using PANTHER classification gene system (release 2015-04-30) with a significant *P* value lower than or equal to 0.05 after Bonferroni correction: (i) among all GO terms associated with *A. fraterculus* and *A. obliqua* CDS transcripts; and (ii) comparing all GO terms of the hybrid transcriptome with the highly differentiated transcripts.

### Mapping reads against a reference transcriptome and SNP discovery

The Bowtie software (version 1.0.0) (Langmead *et al.* 2009) was used to align the Illumina reads back to the transcripts produced by the hybrid assembly. Filtered reads for each species were mapped against the reference transcriptome, generating two reference maps to call SNPs. The resulting bam files were screened for SNPs with SAMtools mpileup (Li *et al.* 2009) with the option that includes a per-sample read depth, and SNPs were recovered with Bayesian inference with bcftools view (Li *et al.* 2009). Transcriptome assemblies include redundant regions in the alternative spliced isoforms. Trinity assembler informs which transcripts came from the same potential gene. We used this information to avoid redundant SNPs, selecting only those from the longest transcript per potential gene, hereafter referred to as unigene. The SNPs were selected using custom scripts, and only those with a minor allele frequency of at least 0.05 per species, minimum Phred-scaled read quality of 30, and minimum read coverage of 100 were retained.

### Differentiation between unigenes of A. fraterculus and A. obliqua

In order to select candidate unigenes showing the highest level of divergence between *A. fraterculus* and *A. obliqua*, we estimated the interspecific differentiation indices *D* and $\bar{D}$ (Renaut *et al.* 2010; Andrés *et al.* 2013) based on the allele frequencies estimated by Samtools using Python scripts. *D* is defined as the absolute value of the difference among the allelic frequencies of a SNP variant of *A. fraterculus* and *A. obliqua* (*D* = |*F*_Af_ − *F*_Ao_|), whereas $\bar{D}$ is the average *D* values for SNPs from a particular unigene. We plotted the distribution of $\bar{D}$, and used $\bar{D}$ ≥ 0.94 as the threshold value to separate the group of most divergent unigenes. We tested normality of the $\bar{D}$ distribution using the Shapiro-Wilk test in the R statistical environment (R Core Team 2015). We also calculated the mean Nei’s population genetic identity and distance (Nei 1972) across all interspecific SNPs, and those SNPs in the selected most divergent unigenes.

### Ka/Ks calculation

Since we sought to identify genes that might be involved in the species differentiation, we estimated the Ka/Ks ratio of nonsynonymous (Ka) to synonymous (Ks) substitutions on the most differentiated unigenes. Because this ratio allows us to evaluate whether the unigenes studied here are evolving under purifying selection (Ka/Ks < 1), neutrally (Ka/Ks = 1), or under positive selection (Ka/Ks > 1), we wanted to separate among the most differentiated unigenes the ones that could have been influenced by selection. To do so, we used the TransDecoder software to generate likely CDSs of the unigenes in the assembly of both species with $\bar{D}$ ≥ 0.94. We compared the selected CDSs against the CDSs predicted from *A. obliqua* and *A. fraterculus* assemblies, and the CDSs of *C. capitata* deposited with GenBank, using the tBLASTx algorithm. Orthologous CDSs were selected using a best reciprocal blast strategy with an *e*-value threshold of < 10^−6^. Each sequence set was translated to amino acids, aligned using the Muscle algorithm (Edgar 2004), then back-translated to the original nucleotide sequence using the program TranslatorX (Abascal *et al.* 2010). We checked all the alignments visually to remove poorly aligned set of sequences. Pairwise Ka/Ks was estimated for each species pair and orthologous set using the Model Selection framework (Posada 2003) by the KaKs Calculator program (Zhang *et al.* 2006), generating then three pairwise Ka/Ks rates per unigene as result, between each pair of species. Genes showing Ka/Ks rates higher than or equal to 0.5 were considered as potentially evolving under positive selection, considering that there might be regions under positive selection amid other more conserved domains that would drive this ratio down (Findlay and Swanson 2010), particularly for recently diverged species. Since our main goal was not only to characterize thousands of new genes in *A. fraterculus* and *A. obliqua*, but particularly to identify genes that, by showing fixed differences between these species, might be involved with their differentiation, we looked for genes that would show low values of Ka/Ks in the branch that connects these species to *Ceratitis* (Ka/Ks < 0.5), but higher rates (Ka/Ks ≥ 0.5) in the branch between *A. fraterculus* and *A. obliqua*, which might suggest that they were evolving under positive selection only between the closely related species pair under study.

### Data availability

The authors state that all data necessary for confirming the conclusions presented in the article are represented fully within the article or available in GenBank with the accession number provided in results.

## Results

### Transcriptome data

The 20 cDNA libraries (five profiles with replicates per species) generated 155,940,826 × 2 raw 100 bp paired-end reads (SRA accession number SRP082299), which were filtered by quality, resulting in 140,493,653 × 2 paired-end reads, and over 28 Gbp (Table 1). The distribution of the raw data generated by Illumina broken down by each library profile by species is shown in Supplemental Material, Table S1. The number of filtered reads, percentage of discarded reads, and number of bases kept in the analysis for each library are shown in Table S2. The libraries had an average of 7 million reads per replicate for *A. fraterculus*, and 6.68 million for *A. obliqua* after quality filtration. We processed these data in Trinity to create a hybrid assembly, which was used as a reference to map the reads of each species and assemblies per species (Table 1). Analyses of differential gene expression among the different species, sex, and developmental stages is presented elsewhere, due to the great number of contrasts performed (F. R. Torres, C. Congrains, E. B. Campanini, S. Chahad-Ehlers, and R. A. de Brito, unpublished data).

**Table 1:** Summary of the sequencing effort, read cleaning and *de novo* assembly statistics of head transcriptomes of adult individuals from both sexes at different life stages of *A. fraterculus* and *A. obliqua*

	*A. fra* + *A. obl**^a^*	*A. fraterculus*	*A. obliqua*
Illumina Sequencing			
Total of paired-end reads	155,940,826	81,781,686	74,159,140
Filtered reads	140,493,653	73,637,239	66,856,414
Assemblies Length Distribution			
Total number of contigs	154,787	112,862	98,549
Number of trinity components	76,293	61,153	55,126
N50	2012	2504	2637
Contigs longer than 1000 bp	45,602	38,105	34,982
Contigs longer than 2000 bp	22,297	21,054	19,964
Contigs longer than 10,000 bp	213	329	269
Assemblies Length Statistics			
Average contig length (bp)	1027	1196	1254
Median contig length (bp)	494	535	563
Longest contig (bp)	25,704	27,513	22,394

a Hybrid assembly generated using reads of head tissues of *A. fraterculus* and *A. obliqua*.

### Functional annotation

TransDecoder predicted 38,522, 34,064, and 45,194 CDSs in the *A. fraterculus*, *A. obliqua* and hybrid assemblies, respectively. We found that 33,209 CDSs of *A. fraterculus* matched with 13,841 CDSs of *D. melanogaster*, 29,894 CDSs of *A. obliqua* matched with 13,794 CDSs of *D. melanogaster*, and 34,341 CDSs of the hybrid assembly matched with 10,961 CDSs of *D. melanogaster*. Annotation against *C. capitata* revealed that 34,556 CDSs of *A. fraterculus* matched with 10,208 CDSs of *C. capitata*, and 31,220 CDSs of *A. obliqua* matched with 10,043 CDSs of *C. capitata*. The software BUSCO identified 1212 single-copy, and 1095 duplicated complete orthologs out of the 2675 genes, and only 244 missing orthologs in the transcriptome of *A. fraterculus*. In *A. obliqua*, BUSCO identified 1236 complete single-copy orthologs, 1071 complete duplicated orthologs, and 250 missing orthologs, whereas the combined assembly identified 1144 single-copy orthologs, 1185 duplicated complete orthologs, and only 216 missing orthologs.

**Figure 2:**
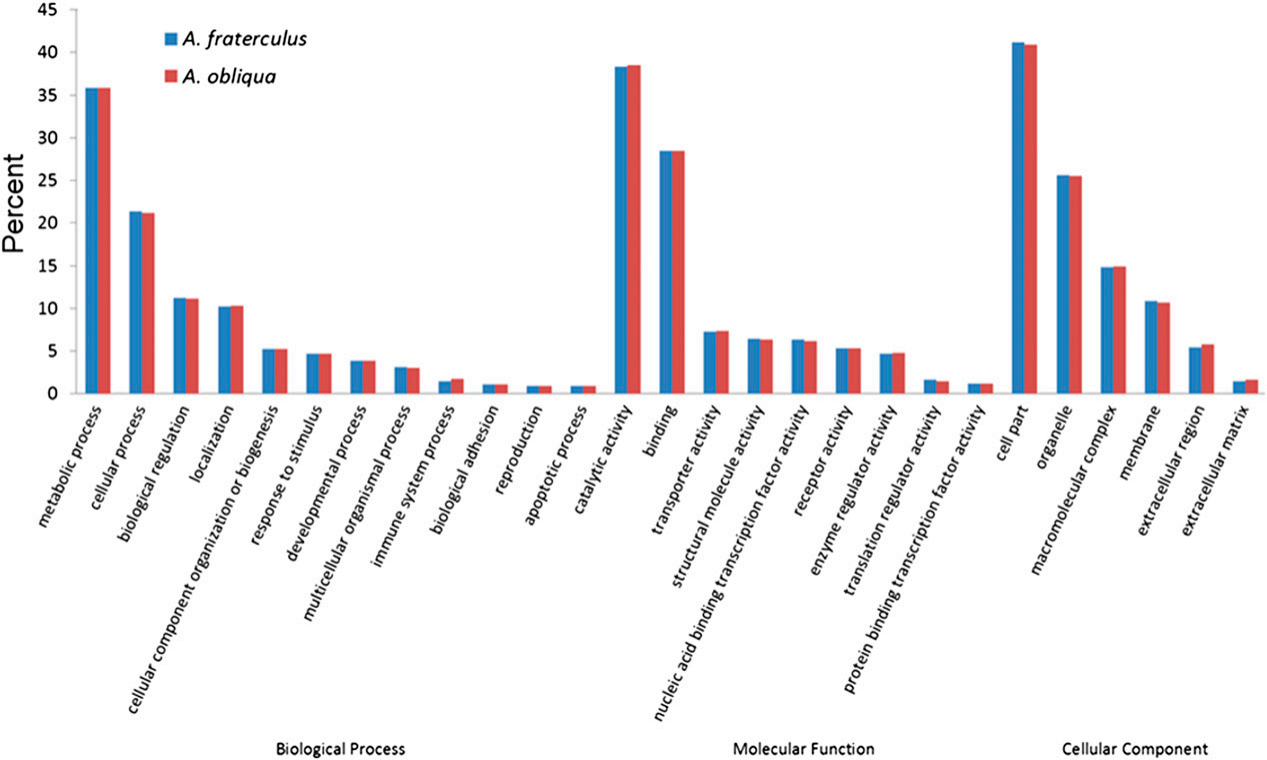
Gene ontology (GO) analysis of the head transcriptomes of *A. fraterculus* and *A. obliqua*. GO terms mapped at the most general level with a percentage > 0.5% are shown.

**Figure 3:**
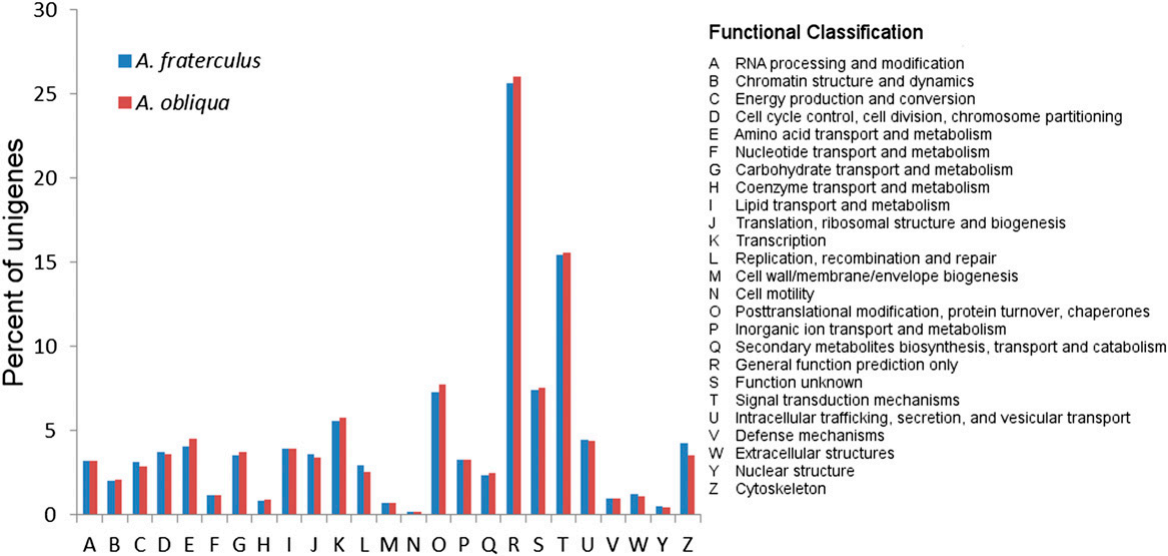
KOG function classification of the head transcriptomes of *A. fraterculus* and *A. obliqua*.

Functional annotation revealed that, among the most general GO terms in biological processes, were metabolic and cellular processes, biological regulation, and localization, whereas binding and catalytic activity were the main ones among molecular function and cell parts, and organelles among the cellular components (Figure 2). Our analysis failed to identify enriched categories distinguishing both species. Totals of 9967 CDSs of *A. fraterculus* and 9354 CDSs of *A. obliqua* were partitioned according to KOG categories (Figure 3). For both species, the largest cluster group represented was general function prediction, followed by signal transduction mechanism (Figure 3).

### SNP discovery and interspecific allele differentiation

**Figure 4:**
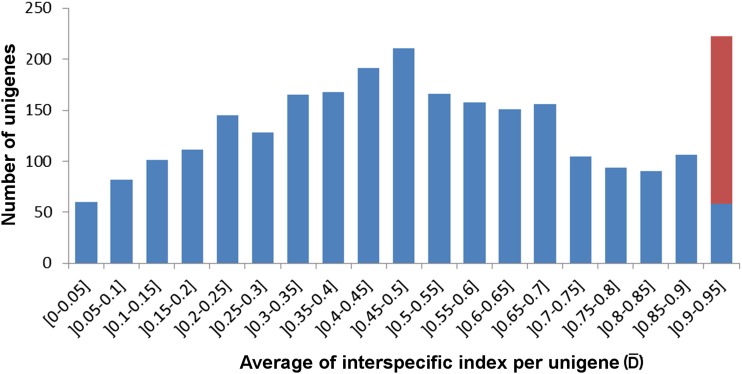
Frequency distribution of $\bar{D}$. Distribution of the average of allele frequency differences between *A. fraterculus* and *A. obliqua* per unigene ($\bar{D}$) in 6386 SNPs across 2612 unigenes. *x*-axis is $\bar{D}$ in intervals of 0.05. Unigenes with the highest differentiation levels (0.94 < $\bar{D}$ ≤ 0.95) are shown in red.

We identified 67,470 SNPs in 5569 unigenes that were specific to *A. fraterculus*, 39,252 SNPs in 4661 unigenes that were limited to *A. obliqua*, and 6386 SNPs, in 2612 unigenes, that were common to both species. We used the differentiation indexes (*D* and $\bar{D}$), that reflect differences in allele frequencies between *A. fraterculus* and *A. obliqua*, to indicate highly differentiated SNPs and unigenes between the species. The distribution of *D* was flat if not for a peak of the class of the most differentiated SNPs (*D* > 0.90) (Figure S1). We identified 647 individual SNPs that showed *D* ≥ 0.94, 0.6% of the total number of SNPs here identified. We considered a threshold of 0.94 for the mean differentiation index ($\bar{D}$) per unigene, which estimated the *D* values of all SNPs per unigene, and identified 164 highly differentiated unigenes that had $\bar{D}$ of at least 0.94. The distribution of $\bar{D}$ had a bimodal distribution, being bell-shaped, with peaks at about 0.5 and 0.95 (Figure 4). Shapiro-Wilks test revealed that the distribution of $\bar{D}$ showed a significant departure from a normal distribution (*w* = 0.9715, *P* < 0.01). The average Nei’s genetic identity and distance across all SNPs was 0.61 and 0.79, respectively, though these values change to 0.053 and 2.93 when only the 214 SNPs of the 164 most differentiated unigenes were considered.

TransDecoder predicted 154 CDSs from the 164 highly differentiated unigenes ($\bar{D}$ ≥ 0.94), and 150 of them showed significant BLAST hits with the database of *D. melanogaster* CDSs. Furthermore, our analysis successfully mapped GO terms for 146 genes. Enrichment analysis showed that 12 GO terms corresponding to cellular component and biological process were over-represented in this set of highly differentiated unigenes (Table 2). We also found that 128 of these unigenes had only one SNP, 28 had two SNPs, and 11 had three or more SNPs. Most of the variants of the eight unigenes with three or more SNPs are in the coding region, but are associated with synonymous substitutions (Table 3).

**Table 2:** Functional enrichment of highly differentiated unigenes ($\bar{D}$≥ 0.94)

GO term	Category	Main Category*^a^*	Number of Genes	Fold Enrichment	*P*-Value*^b^*
GO:0005737	Cytoplasm	CC	64	1.65	3.23E−03
GO:0044444	Cytoplasmic part	CC	51	1.76	9.74E−03
GO:0005829	Cytosol	CC	18	2.86	3.81E−02
GO:0005622	Intracellular	CC	90	1.35	3.89E−02
GO:0044237	Cellular metabolic process	BP	70	1.61	7.82E−03
GO:0006412	Translation	BP	16	3.87	1.13E−02
GO:0006518	Peptide metabolic process	BP	17	3.58	1.57E−02
GO:0043043	Peptide biosynthetic process	BP	16	3.77	1.58E−02
GO:1901566	Organonitrogen compound biosynthetic process	BP	21	2.96	2.21E−02
GO:0008152	Metabolic process	BP	81	1.47	2.34E−02
GO:0043604	Amide biosynthetic process	BP	16	3.64	2.41E−02
GO:0043603	Cellular amide metabolic process	BP	17	3.33	3.98E−02

a CC, cellular component; BP biological process.

b *P*-values were corrected using a Bonferroni approach.

**Table 3:** Highly divergent unigenes with at least three SNPs

*Drosophila* CDSs Database Annotation	$\bar{D}$	*D*	Amino Acid Involved		Substitution Type*^a^*
Thioredoxin reductase-1	0.950	0.950	Arg (R)	Lys (K)	NS
		0.950	Asn (N)	Asn (N)	S
		0.950	Ser (S)	Leu (L)	NS
Maternal expression at 31B	0.950	0.950	Val (V)	Val (V)	S
		0.950	Ile (I)	Ile (I)	S
		0.950	Ala (A)	Ala (A)	S
		0.950	Ala (A)	Ala (A)	S
Superoxide dismutase	0.950	0.950	—	—	NC
		0.950	Thr (T)	Thr (T)	S
		0.950	Thr (T)	Thr (T)	S
CG3842	0.950	0.950	Ser (S)	Ser (S)	S
		0.950	Gly (G)	Gly (G)	S
		0.950	Ala (A)	Ala (A)	S
		0.950	Arg (R)	Arg (R)	S
		0.950	Leu (L)	Ile (I)	NS
CG2233	0.942	0.919	Gly (G)	Glu (E)	NS
		0.950	Asn (N)	Asp (D)	NS
		0.950	Ser (S)	Ser (S)	S
		0.950	Leu (L)	Leu (L)	S
CG32425	0.950	0.950	Ile (I)	Ile (I)	S
		0.950	Asn (N)	Asn (N)	S
		0.950	—	—	NC
Flavin-containing monooxygenase 2	0.950	0.950	Ser (S)	Thr (T)	NS
		0.950	Glu (E)	Glu (E)	S
		0.950	Gly (G)	Gly (G)	S
		0.950	Val (V)	Val (V)	S
		0.950	Asn (N)	Asn (N)	S
Relish	0.949	0.949	Asp (D)	Glu (E)	NS
		0.950	Leu (L)	Leu (L)	S
		0.949	Arg (R)	Arg (R)	S

$\bar{D}$ is average of the interspecific index (*D*) between *A. fraterculus* and *A. obliqua* calculated for all SNPs of each unigene

a NC, Substitution in noncoding region; N, nonsynonymous substitution; S, synonymous substitutions.

### Selective pressure of highly differentiated unigenes

The 164 most differentiated unigenes between species were submitted to a Ka/Ks analysis by identifying each ortholog CDS, and aligning it to the corresponding ortholog CDSs of *A. fraterculus*, *A. obliqua*, and *C. capitata*, but only 150 of these had potential orthologs across *C. capitata*, and for one we could not find orthologs between *A. fraterculus* and *A. obliqua*. Two sets of CDSs were poorly aligned, and were removed from further analyses. Hence, we performed the Ka/Ks analysis on 148 potential orthologs that were adequately aligned among *C. capitata*, *A. fraterculus*, and *A. obliqua*, which allowed us to identify 11 unigenes with Ka/Ks > 0.5 (Table 4). All unigenes that showed high values of Ka/Ks between *A. obliqua* and *A. fraterculus* also showed high values when these unigenes were contrasted with *C. capitata*. SNP calling analysis revealed that two of these highly differentiated unigenes had a SNP in the 3′ UTR, whereas nine unigenes showed SNPs in the coding region, seven of which associated with nonsynonymous substitutions, and two with synonymous substitutions (Table 4).

**Table 4:** Highly divergent unigenes evolving under positive selection in the *Anastrepha* branch

*Drosophila* CDSs Database Annotation	SNP Calling				Pairwise Ka/Ks*^b^*		
	$\bar{D}$	Amino Acid Involved		Substitution Type*^a^*	*A. fra* × *A. obl*	*A. obl* × *C. cap*	*A. fra* × *C. cap*
Ribosomal protein L24-like	0.950	—	—	NC	50.0000(0.002/4.5E^−05^)	0.0188(0.024/1.257)	0.0168(0.021/1.272)
Serine protease 6	0.950	Gln	Gln	S	1.2455(0.023/0.019)	0.2743(0.601/2.189)	0.2903(0.607/2.092)
CG16817	0.949	Ser	Arg	N	0.7325(0.007/0.009)	0.0830(0.152/1.835)	0.0762(0.151/1.988)
CG2219	0.950	Ser	Thr	N	0.7302(0.009/0.013)	0.1050(0.235/2.239)	0.1073(0.289/2.697)
CG13367	0.949	Ser	Pro	N	0.7168(0.002/0.003)	0.0826(0.168/2.034)	0.0838(0.167/1.997)
Microtubule-associated protein 205	0.949	Glu	Glu	S	0.6091(0.018/0.030)	0.2762(0.321/1.162)	0.2852(0.325/1.139)
Odorant-binding protein 99c	0.950	Glu	Ile	N	0.6038(0.030/0.049)	0.1401(0.124/0.887)	0.1281(0.122/0.953)
CG9500	0.950	Glu	Ala	N	0.6025(0.036/0.059)	0.1160(0.353/3.042)	0.0869(0.337/3.877)
		Glu	Ala	N
Ribonuclear protein at 97D	0.950	—	—	NC	0.5627(0.017/0.031)	0.0243(0.044/1.822)	0.0225(0.040/1.767)
Transferrin 3	0.943	Glu	Ser	N	0.5614(0.007/0.012)	0.0522(0.088/1.684)	0.0528(0.088/1.674)
Mitochondrial ribosomal protein S2	0.950	Thr	Thr	S	0.5462(0.010/0.017)	0.0311(0.083/2.676)	0.0344(0.083/2.425)

$\bar{D}$ is the interspecific index between *A. fraterculus* and *A. obliqua* calculated for each transcript. Amino acid substitution is associated to the SNP analysis. *A. fra*, *A. fraterculus*; *A. obl*, *A. obliqua*; *C. cap*, *C. capitata*.

a NC, Substitution in noncoding region; N, nonsynonymous substitution; S, synonymous substitution.

b Values of Ka/Ks rates and values of Ka and Ks, separated by a slash, are shown in parentheses.

## Discussion

### Transcriptomes generated from head tissue of A. fraterculus and A. obliqua

Transcriptomes are very dynamic due to the inherent plasticity in gene expression in response to changes to several genetic and environmental conditions. Aiming at producing transcriptomes from a large array of genes expressed in two closely related species, *A. fraterculus* and *A. obliqua*, we sampled tissues from both sexes at different life stages. Since the assembly is a critical step, the large raw dataset generated here was checked carefully for adaptors and quality of the reads, especially because we lack a reference genome for the species here investigated, or from any species in the genus *Anastrepha* that could help us assess rates of erroneous contig inferences. We generated three assemblies, one for each species, and a hybrid that was used as a reference for SNP inference. *A. obliqua* and *A. fraterculus* are phylogenetically close, so we expected that the repertoire of expressed transcripts would be similar, and the level of genetic distance of the majority of transcripts in both species would be low. To evaluate that, we needed to assess the general quality of the assemblies. We first considered the N50 parameter under the justified assumption that gene length is usually well conserved between related species. We found that the N50 values of the three assemblies were similar, though the hybrid assembly had values slightly lower than the other two, possibly a consequence of the higher number of contigs in the hybrid assembly, but possibly also be due to the increased heterogeneity brought by the combination of the two species. It is noteworthy that the combined assembly generated between 20% and 30% more transcripts longer than 1000 bp than the individual ones. A comparison of the N50 indices inferred for the three assemblies generated here are in line with the values obtained for other insect transcriptomes (Crawford *et al.* 2010; Garg *et al.* 2011; Salvemini *et al.* 2014).

N50 should be interpreted carefully because it suffers from the fact that its measure is based on the distribution of contig sizes, rather than on their accuracy (Li *et al.* 2014; Miller *et al.* 2010). Depending on the conditions of the assembly, longer chimeric contigs may be produced that bear no proximity with real transcripts. Therefore, we considered other strategies to investigate the accuracy of the assemblies, particularly the similarity to groups of conserved genes. One strategy we used was to compare the ORFs inferred from the each assembly against a set of genes inferred by BUSCO, which is composed of a long list of conserved orthologs derived from OrthoDB, considering a phylogenetically sound inference (Simão *et al.* 2015). Based on the comparison of the transcriptomes with BUSCO arthropod set, we recovered an average of 86% of complete genes in all assemblies, 5% fragmented, and only about 9% missing. These results corroborate the data from CEGs to indicate that all assemblies have a high level of completeness, and suggest that the hybrid assembly had slightly more complete orthologs than each individual one (it missed on average 15% less orthologs than each individual assembly), but also produced more duplicates, probably a consequence of heterogeneity across transcripts from different species. Therefore, the hybrid transcriptome used reflected the particularities of each species and provided more completeness of the shared transcripts, but it did not introduce significant noise to limit its applicability. We should consider that these type of comparisons were aimed at evaluating the completeness of genome projects (Simão *et al.* 2015), so we do not necessarily expect all the genes that were present in the conserved set to be expressed in all tissues and life stages of the species under study. Therefore, the fact that we recovered close to 90% of BUSCO genes, conserved or otherwise, in all these contrasts, is highly significant, and indicates that the transcriptomes assembled here are fairly complete, particularly when you consider that they were generated from a specific tissue. Because BUSCO focuses on conserved genes, it would be a fairer representation of the completeness of the transcriptome than a contrast with the whole transcriptome, since the latter might be influenced by fast-evolving genes that would drive these values down.

We also performed BLASTx searches against other important databases. A contrast against NCBI nr-database reveals that ∼99% of the best hits were against insect genes for all assemblies, the great majority against Tephritidae sequences, reiterating the quality of these transcriptomes. A small proportion of transcripts, on average 5% per assembly, failed to produce significant hits against the nr-database. We also identified sequences of *Wolbachia*, which has already been described to infect populations of different species of *Anastrepha*, among them *A. fraterculus* and *A. obliqua* (Coscrato *et al.* 2009). We also contrasted our sequences against *D. melanogaster*, because it is the best curated insect genome. Based on this comparison, we found that 86% of the CDSs predicted for *A. fraterculus*, and 87.8% for *A. obliqua* matched with CDSs of the more distantly related *D. melanogaster*.

We used GO annotation to investigate the functional attributes of the transcriptomes here assembled, allotting the translated inferred ORFs produced into three different GO ontologies, biological process, molecular function, and cellular component, which are derived from a comprehensive description of each gene properties and their products in any organism. The distribution of GO terms was very similar between *A. fraterculus* and *A. obliqua*, so much so that an enrichment test performed on PANTHER classification gene system failed to identify significantly enriched categories between these assemblies. The obtained distributions of GO terms are similar to other distributions previously published for other insects, though not necessarily for the same tissues (Shen *et al.* 2011; Salvemini *et al.* 2014; Van Belleghem *et al.* 2012; Wang *et al.* 2010). Likewise, our analyses failed to detect significant differences when comparing the distribution of COG classes between *A. fraterculus* and *A. obliqua*.

### Screening for highly differentiated genes

Some species in the *fraterculus* species group are closely related, and show limited genetic and morphologic differentiation (Smith-Caldas *et al.* 2001; Araujo 1997; Zucchi 2000a), in part because they have diverged recently. Furthermore, there is evidence that these species are able to successfully mate in the lab (Henning and Matioli 2006; Abraham *et al.* 2011; Aluja *et al.* 1999), and even that they differentiated with introgression (F. Diaz, A. L. A. Lima, A. M. Nakamura, F. Fernandes, I. Sobrinho, and R. A. de Brito, unpublished results). Hence, it was not surprising that we failed to identify categories of genes that differ between *A. obliqua* and *A. fraterculus*. Our strategy then focused on identifying SNPs and unigenes that would show high levels of polymorphism, and, more specifically, species-specific fixed differences that could have been affected by natural selection, which had hitherto not been identified for these species. The fact that 64% more SNPs were identified in *A. fraterculus* than in *A. obliqua* may be a consequence of larger population sizes or higher heterogeneity in *A. fraterculus*, so much so that it is considered to be a species complex (Hernández-Ortiz *et al.* 2012), but could also be due to particularities of the populations from which these species were sampled.

We should point out that our analysis considered as SNPs only sites in which the rarest allele had a frequency of ≥ 0.05, so it is possible that there are more shared polymorphisms between the species than was accounted for. However, by not considering low frequency SNPs, we avoided potential errors generated from the next generation sequencing. A robust threshold (≥0.94) was chosen for the interspecific differentiation indexes (*D* and $\bar{D}$) to select only unigenes with alternative nucleotides practically fixed between *A. fraterculus* and *A. obliqua*. Because we considered only a single population per species for our analysis, it is possible that these estimates may have failed to consider within-species variation, but other studies have reported low levels of fixed intraspecific variation across populations in species of the *fraterculus* group, and no species-specific fixed polymorphism for the species studied here has been reported (Gonçalves *et al.* 2013; Smith-Caldas *et al.* 2001; Barr *et al.* 2005). So, our main goal was to identify highly differentiated unigenes with fixed differences between species that could be considered as markers to be used to identify these species, as well as potential candidate genes to be involved in the species’ differentiation, not an overall estimate of variability for the species, which would require the use of fine scale strategies, including geographically widespread samples.

The class of 164 highly differentiated unigenes represents a departure from the normal distribution of average $\bar{D}$ (Figure 4), and the first set of fixed species-specific differences between the species here studied, but to distinguish SNPs that were possibly independently fixed by drift from those that differentiated in response to selection, we investigated whether these unigenes showed higher levels of Ka/Ks. We searched for signals of selection initially by looking at some patterns that would unify this class, such as patterns of distribution in GO categories, and we identified a significant enrichment of two metabolic processes: translation and biosynthesis of peptides (Table 2). Even though it is difficult to establish a direct relationship between differences in fitness and protein synthesis, diets rich in protein stimulate reproductive success in tephritids because of their impact on female’s oviposition rate (Oviedo *et al.* 2011), so selection to different diets may drive these genes apart. It is noteworthy that these GO terms encompass several ribosomal proteins, three of which are candidate genes to be involved in the species differentiation because they had highly differentiated SNPs and an average Ka/Ks > 0.5 (Table 4).

Furthermore, since the class of highly differentiated contigs harbors the most difference between the species, the genetic identity between species estimated by the SNPs is close to zero, whereas, overall, the genetic identity is 0.61, indicating that most SNPs shared a substantial proportion of alleles. Likewise, Nei’s genetic distance is almost four times higher in SNPs located in highly differentiated unigenes, which suggests that some variants in these genes might lead the divergence between these closely related species. The majority of unigenes in this class harbor only a single highly differentiated SNP, though eight had three or more (Table 3). We considered these eight unigenes candidate genes to be involved in the differentiation between *A. fraterculus* and *A. obliqua* because, when the low interpopulation diversity and recent divergence time between the species is taken into account, this large number of fixed differences in the same unigene may be an indication that there was strong selection favoring the fixation of different copies on different species. Though in most cases these SNPs are involved with changes that do not lead to amino acid changes, they could have been driven to fixation by a strong selective sweep on variants to which they are linked, indicating a relevant role for selection at or near these unigenes.

To investigate whether selection could be associated with the evolution of these 164 highly differentiated unigenes, we looked for their patterns of synonymous and nonsynonymous changes, and identified 11 unigenes with a Ka/Ks cutoff value of ≥ 0.5 (Table 4). Though technically the value of Ka/Ks should exceed 1.0 for positive selection to be considered, the majority of genes show regions under positive selection amid other more conserved domains, because most genes retain relevant activities in the organism (Findlay and Swanson 2010). So even when these genes are under positive selection, it is likely that this would occur only at some portions of the gene, and there will be other conservative domains that would drive down the Ka/Ks ratio. It has been suggested that a value of 0.5 might be adequate to indicate that the gene may have portions under positive selection, while having others under purifying selection (Swanson *et al.* 2004). Furthermore, when species have diverged recently, there is a wider amplitude in this estimate (Keightley and Eyre-Walker 2012) that limits the efficacy of the use of such estimates for intra and interpopulation data. Because the purpose here is more to identify potential genes that would be involved with species differentiation, we used a less conservative approach, considering that any corroboration would demand a more strict evaluation. All but one of the 11 unigenes with a Ka/Ks ≥ 0.5 (Table 4) were associated with only a single highly differentiated SNP. However, the majority of changes were associated with nonsynonymous substitutions, which, by promoting amino acid changes, may promote important changes on protein function, and even lead to drastic phenotypic consequences (Ng and Henikoff 2006). Tests that evaluated whether these changes led to radical or conservative amino acid changes found such changes in ≥ 10 of the genes here studied (data not shown), indicating the potential for some changes that drastically alter protein structure. Some SNPs are associated with synonymous changes that do not lead to amino acid changes, but may still lead to differential selection, particularly when you consider the existence of codon bias (Andolfatto 2005; Suzuki and Gojobori 1999; Resch *et al.* 2007), which we failed to find in *A. fraterculus* and *A. obliqua* (Nc indices were around 55 for both species). Furthermore, we also failed to find significant differences in codon usage between the species (mean square difference = 0.002, n.s.). We also considered these 11 unigenes candidate genes involved in differentiation of the *fraterculus* group.

Therefore, our strategy led to 19 transcripts representing potential candidate genes either because they were associated with Ka/Ks > 0.5 or had a large number of fixed species-specific SNPs, suggesting, in both instances, that selection may have driven these gene’s differentiation. Though we will not discuss specifics for all candidate genes identified here, we could distinguish two main targets that encompass almost half of the candidate genes: oxidative stress and odorant receptors, both of which that could have major implications for the speciation processes in these flies.

Four of the candidate genes are involved in the regulation of intracellular redox homeostasis and oxidative stress: thioredoxin reductase-1 (*Trxr-1*), superoxide dismutase, flavin-containing monooxygenase-2, and *CG3842*. Though *CG3842* has not been well characterized, its structure suggests oxidoreductase activity, catalyzing a redox reaction in which a CH–OH group acts as an electron donor. Cellular antioxidant enzymes play crucial roles in aerobic organisms by inactivating potentially damaging oxygen agents, which can range from specific amino acid modifications to total enzyme inactivation (Stadtman 1986), and can also lead to DNA modifications and cross-linkage of proteins (Imlay 2003). Enzymes involved with oxidative stress have been implicated in many essential life history traits, such as reproduction, senescence (Tower 2015; Orr and Sohal 1994), and longevity (Buttemer *et al.* 2010), as well as to many other important stress related factors, such as extreme temperatures (Storey and Storey 2010; Lalouette *et al.* 2011; Williams *et al.* 2014), desiccation (Benoit 2010), hypoxia, and anoxia (Azad *et al.* 2009; Zhao and Haddad 2011; Benasayag-Meszaros *et al.* 2015), which can be very common during larval development depending on fruit condition.

Another candidate gene, *Relish*, is a member of the REL family of proteins that has been shown to be overexpressed in hypoxia (Liu *et al.* 2006), though it is also involved with several cellular and organismal processes, such as immunity and embryogenesis (Dushay *et al.* 1996). A study in this gene on two recently diverged species, *D. simulans* and *D. melanogaster*, found strong evidence for adaptive protein evolution in the former, but not in the latter (Begun and Whitley 2000). Several genes that have been shown to be involved with hypoxia are, in fact, associated with other stress pathways, such as heat shock proteins, hinting that the physiological response to hypoxia and other stresses taps into similar multiple stress response pathways (Liu *et al.* 2006), reinforcing the potential relevance of *CG16817*, identified as being under positive selection, which belongs to the p23/wos2 family of chaperones that have been found to be involved with other heat shock proteins in the folding of regulatory proteins (Garcia-Ranea *et al.* 2002), and are part of the primary response to stress.

Response to odors might have an important role in speciation, particularly in fruit flies, so it is relevant that we found evidence of positive selection acting on different genes in the reception and processing of odors. One of these genes was *Maternal expression 31B* (*Me31B*), which codes for a member of the DEAD box helicase family, and, in *Drosophila*, is a translation repressor protein (Hillebrand *et al.* 2010) that regulates embryonic patterning, but also controls miRNA expression (Barbee *et al.* 2006; Chu and Rana 2006) and regulation of a *CaMKII* mRNA reporter in dendritic elements of olfactory sensory and projection neurons. Projection neurons in the antennal lobes of *D. melanogaster* indicate rates of change of odor stimuli (Kim *et al.* 2015), fostering the onset of response to odors, so the control of *CaMKII*, which is a protein kinase associated with memory formation (Malik *et al.* 2013), olfactory control (Akalal *et al.* 2010), and even long-term memory of courtship rejection in *D. melanogaster* (Winbush *et al.* 2012), could have important implications for odor recognition and memory. Furthermore, we also identified, *Olfactory binding proteins* (*OBPs*), which are the first component of the insect olfactory system, solubilizing and carrying the chemical signals from the environment to the odorant receptors (Sanchez-Gracia and Rozas 2008). There is evidence that positive selection has been involved in the evolution of the *OBP* gene family in insects (Forêt and Maleszka 2006; McBride and Arguello 2007; Vieira *et al.* 2007; Gotzek *et al.* 2011), suggesting that changes in *OBP*s may result in changes in olfactory behaviors. We found positive selection in the *OBP99c* gene in the comparison between *A. fraterculus* and *A. obliqua*, but not between any *Anastrepha* and *C. capitata*, which could be because they have accumulated sufficient changes to hamper the signal of positive selection in this comparison. The percentage of divergence between the two *Anastrepha* species for this *OBP* is 3.2%, whereas the average divergence between *OBP99c* from these species and *C. capitata*’s is ∼25%. This level of divergence may not be sufficient to lead to overall homoplasy, but it may lead to homoplasy in the regions that are evolving more rapidly, which could explain the fact that we failed to detect positive selection between *Anastrepha* and *Ceratitis*. This *OBP* (as well as *OBP99a* and *OBP99d*) is responsible for recognizing benzaldehyde (Wang *et al.* 2007)—an aromatic compound that is associated with several fruits (Rodríguez *et al.* 2013)—so different *OBPs* may lead to adaptation to different host plants. Even though *A. fraterculus* and *A. obliqua* are somewhat generalists, they tend to have different host preferences, with the former being more adapted to Myrtaceae, whereas the latter prefers Anacardiaceae, though they are commonly found in both, it is possible that they have experienced different ecological constraints since the separation of these two species. Because we used mangoes to replicate populations from both species in the lab, it is possible that this adaptation could have happened after these populations have been transferred to the lab, since it has been suggested that such adaptations may happen in a single generation (Christie *et al.* 2012). We do not believe this to be the case, because adaptation to captivity depends not only on strong differential selection compared to wild populations, but also on the population sizes and number of generations in the lab (Frankham 2008). The populations studied here were kept in the lab in large population sizes for no longer than four generations, and the species studied here are commonly found in mangoes (Nascimento *et al.* 1992).

It has been suggested that the speciation process might be driven by some key genes, whereas the rest of the genome would be somewhat porous, and might possibly even experience gene flow (Feder *et al.* 2013; Wu 2001). Through time these so called islands of speciation would grow into continents, limiting the gene flow to smaller and smaller portions of the genome, until the point of complete reproduction isolation is reached (Nosil and Feder 2012; Turner *et al.* 2005). This view has been questioned because a similar pattern in which areas in the genome with high differentiation interspersed with low differentiated areas could also be explained simply by natural or sexual selection, and even the vagaries of the drift process acting on specific genes separated by somewhat neutral regions (Noor and Bennett 2009; Turner and Hahn 2010). In this case, there would be a combination of regions with high levels of differentiation on the genome, driven by selection, or even a postspeciation adaptation such as a Dobzhansky-Muller process, or drift, and others that would still segregate ancestral polymorphism even in the absence of gene flow (Cruickshank and Hahn 2014). The main differences between these two models stem not only from the potential existence of gene flow, but also that these regions of high divergence in one model would be the driving force behind the speciation process, whereas in the other model, though they could be important for speciation, they could also be a secondary adaptation after the speciation, for instance, when there is specialization to different niches, or even be simply a consequence of the overdispersion of fixation driven by drift. The abundat existence of shared polymorphisms suggests that they are probably distributed throughout the genome, though we still lack genomic information on these species to verify that. The reduced number of fixed species-specific differences between *A. fraterculus* and *A. obliqua* confirms that their divergence is very recent, and still limited to few genes, but does not tell us if the reduced number of SNPs that are highly differentiated are actually involved in species differences. Even though the indication of positive selection acting on some of these regions, particularly associated with oxidative stress and olfactory receptors, could indicate their importance in the species’ divergence, we should point out that, in general, our results failed to find strong evidence of selection acting on the transcriptome of these species, which could be due to the difficulty to detect selection driving species that diverged so recently, but it could also indicate that their differentiation could have been driven by drift, rather than selection.

### Conclusions

The species studied here are very closely related, so much so that, hitherto, there was no evidence of fixed differences between them, with evidence of divergence with introgression. Here, we identified at least 647 SNPs that might be fixed between these species, as well as putative genes that showed patterns of molecular changes consistent with changes driven by positive selection that might be involved with species differences in the *fraterculus* group. In spite of that, our results failed to detect regions in the genome that have shown strong evidence of positive selection, so we cannot refute the possibility that divergence between these species has been driven by stochastic processes rather than selection. Therefore, we have moved one step closer to our quest of identifying genes involved in species differences in this important group of fruit flies by identifying a set of SNPs and genes that show fixed species-specific differences between *A. fraterculus* and *A. obliqua*. We should now confirm the potential of these candidate genes not only as species-specific markers when considering other populations, but also the potential for these genes to help us understand processes affecting other species in the *fraterculus* group, as well as other *Anastrepha*.

## Supplementary Material

Supplemental Material
