# Supplementary material for: Head Transcriptomes of Two Closely Related Species of Fruit Flies of the Anastrepha fraterculus Group Reveals Divergent Genes in Species with Extensive Gene Flow
Source: G3 (Bethesda). 2016 Aug 23;6(10):3283–95. doi: 10.1534/g3.116.030486 (PMC5068948; doi:10.1534/g3.116.030486)
Supplement: Supplemental Material [file supp_6_10_3283__index.html]

Head Transcriptomes of Two Closely Related Species of Fruit Flies of the Anastrepha fraterculus Group Reveals Divergent Genes in Species with Extensive Gene Flow — Head Transcriptomes of Two Closely Related Species of Fruit Flies of the Anastrepha fraterculus Group Reveals Divergent Genes in Species with Extensive Gene Flow — Supplemental Material 

# Head Transcriptomes of Two Closely Related Species of Fruit Flies of the *Anastrepha fraterculus* Group Reveals Divergent Genes in Species with Extensive Gene Flow

## Supplemental Material for Resende *et al.*, 2016

**Files in this Data Supplement:**

- Figure S1 - Frequency distribution of D. Distribution of allele frequency differences among 6,386 shared SNPs of *A. fraterculus* and *A. obliqua*. X- axis is D in intervals of 0.05. SNPs with the highest differentiation levels (D > 0.9) are showed in red. (.pdf, 66 KB)
- Table S1 - Sequencing effort per library. (.pdf, 92 KB)
- Table S2 - Total number of filtered reads, percentage of reads retained and total number of bases produced per library and per species. (.pdf, 106 KB)
