# Supplementary material for: Head Transcriptomes of Two Closely Related Species of Fruit Flies of the Anastrepha fraterculus Group Reveals Divergent Genes in Species with Extensive Gene Flow
Source: G3 (Bethesda). 2016 Aug 23;6(10):3283–95. doi: 10.1534/g3.116.030486 (PMC5068948; doi:10.1534/g3.116.030486)
Supplement: Supplemental Material [file supp_g3.116.030486_FigureS1.pdf]

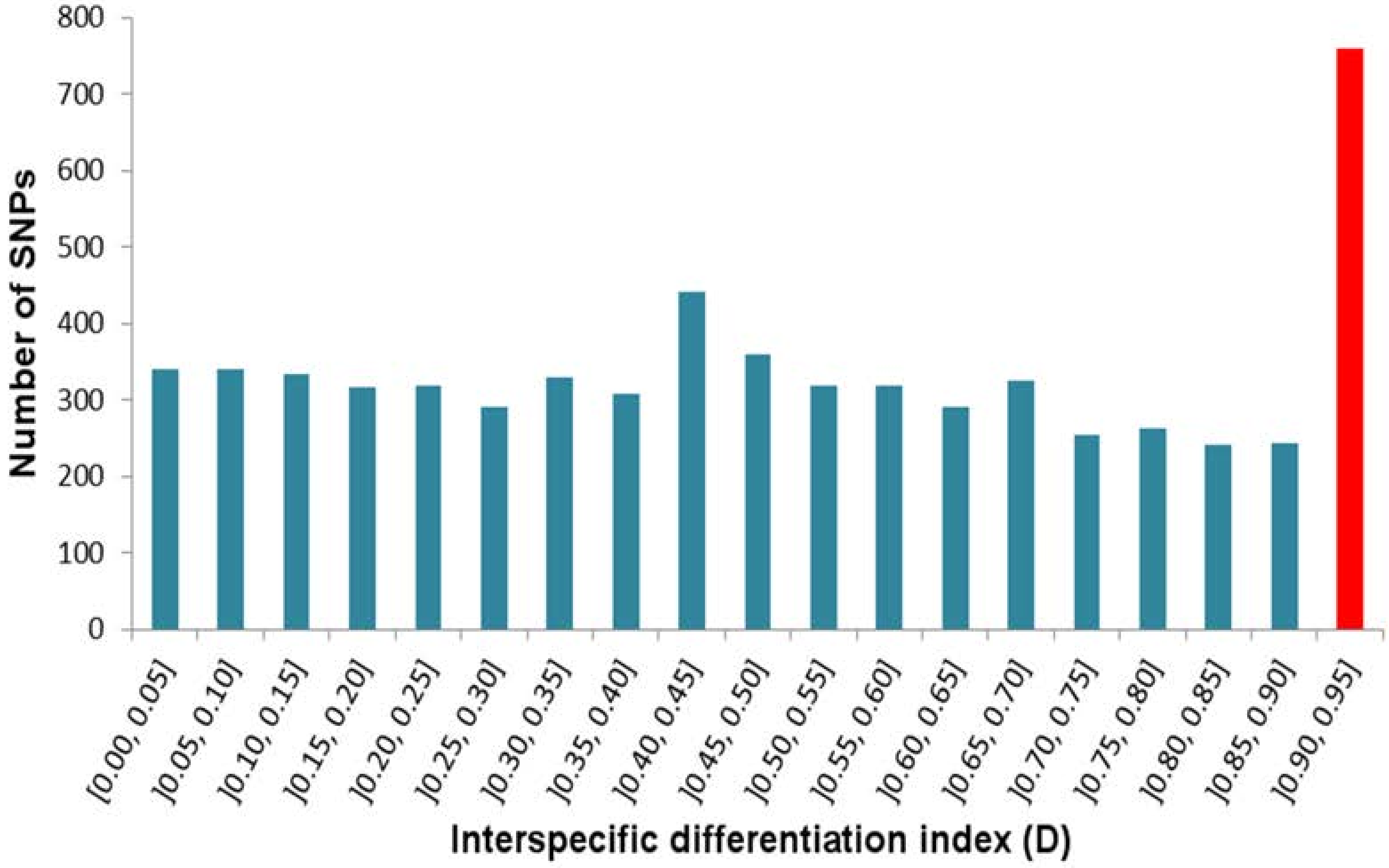

Figure S1. Frequency distribution of D. Distribution of allele frequency differences among 6,386 shared SNPs of *A. fraterculus* and *A. obliqua*. X- axis is D in intervals of 0.05. SNPs with the highest differentiation levels (D > 0.9) are showed in red.
