# Supplementary material for: Head Transcriptomes of Two Closely Related Species of Fruit Flies of the Anastrepha fraterculus Group Reveals Divergent Genes in Species with Extensive Gene Flow
Source: G3 (Bethesda). 2016 Aug 23;6(10):3283–95. doi: 10.1534/g3.116.030486 (PMC5068948; doi:10.1534/g3.116.030486)
Supplement: Supplemental Material [file supp_g3.116.030486_TableS1.pdf]

Table S1. Sequencing effort per library.

Libraries Profile		Replicates	Reads	
			A. fraterculus	*A. obliqua*
Female	Virgin	A	6,500,046	7,497,373
		B	6,822,474	6,891,525
	Post mating	A	7,183,605	8,335,243
		B	9,149,410	6,710,548
	Post oviposition	A	6,531,891	8,208,959
		B	9,420,206	8,649,653
Male	Virgin	A	7,736,532	6,195,410
		B	8,683,757	7,021,347
	Post mating	A	11,364,858	7,840,733
		B	8,388,907	6,808,349
Total by species			81,781,686	74,159,140
Total			155,940,826	
