# Supplementary material for: Head Transcriptomes of Two Closely Related Species of Fruit Flies of the Anastrepha fraterculus Group Reveals Divergent Genes in Species with Extensive Gene Flow
Source: G3 (Bethesda). 2016 Aug 23;6(10):3283–95. doi: 10.1534/g3.116.030486 (PMC5068948; doi:10.1534/g3.116.030486)
Supplement: Supplemental Material [file supp_g3.116.030486_TableS2.pdf]

Table S2. Total number of filtered reads, percentage of reads retained and total number of bases produced per library and per species.

Libraries		Rep.	*A. fraterculus*						*A. obliqua*					
			**Reads**	**%**	**Bases**	**%**	**PE1***	**PE2***	**Reads**	**%**	**Bases**	**%**	**PE1***	**PE2***
Female	Virgin	A	5,652,273	86.95	1,086,767,944	83.59	96	96	6,684,039	89.15	1,278,769,665	85.28	95	95
		B	6,280,265	92.05	1,205,934,440	88.37	95	96	6,171,699	89.55	1,178,119,819	85.47	95	95
	Post mating	A	6,186,816	86.12	1,173,833,135	81.70	97	92	7,520,056	90.22	1,436,357,133	86.16	95	95
		B	8,519,269	93.11	1,647,889,520	90.05	96	96	6,066,139	90.39	1,152,296,056	85.85	94	95
	Post oviposition	A	5,492,619	84.08	1,034,443,269	79.18	97	91	7,345,410	89.48	1,406,454,916	85.66	95	95
		B	8,773,354	93.13	1,697,983,441	90.12	96	96	7,811,619	90.31	1,492,531,036	86.27	95	95
Male	Virgin	A	7,241,354	93.59	1,401,642,013	90.58	96	96	5,661,286	91.37	1,090,347,302	87.99	96	96
		B	8,021,534	92.37	1,548,755,751	89.17	96	96	6,422,330	91.46	1,236,636,692	88.06	96	96
	Post mating	A	9,663,362	85.02	1,827,937,120	80.42	97	92	7,004,874	89.33	1,340,493,523	85.48	95	95
		B	7,806,393	93.05	1,510,592,958	90.03	96	96	6,168,962	90.60	1,186,715,711	87.15	96	96
Total by species			73,637,239		14,135,779,591				66,856,414		12,798,721,853			

* Average size by sequence reading of paired-end (PE).
